# The impacts of infectious complications on outcomes in acute pancreatitis: a retrospective study

**DOI:** 10.1186/s40779-020-00265-5

**Published:** 2020-08-16

**Authors:** Xun Jiang, Ji-Yu Shi, Xia-Yu Wang, Yong Hu, Yun-Feng Cui

**Affiliations:** 1 Tianjin Medical University, Tianjin, 300041 China; 2 Department of Surgery, Tianjin Nankai Hospital, Nankai Clinical School of Medicine, 122 Sanwei Road, Nankai district, Tianjin, 300110 China

**Keywords:** Acute pancreatitis, Infectious complications, Extrapancreatic infection, Mortality

## Abstract

**Background:**

The occurrence of infectious complications characterizes the more severe forms of acute pancreatitis (AP) and is associated with high mortality. We investigated the effects of infection at different sites in patients with AP, including those with necrotizing pancreatitis (NP).

**Methods:**

We conducted a retrospective analysis of 285 patients who met the inclusion criteria for AP and were admitted to Tianjin Nankai Hospital between January 2016 and September 2019. According to the source of the culture positivity during hospitalization, patients were divided into four groups: sterile group(*n* = 148), pancreatic infection group(*n* = 65), extrapancreatic infection group(*n* = 22) and combined infection group(*n* = 50). The source of infection, microbiology, biochemical parameters and prognostic indicators were analyzed.

**Results:**

In terms of baseline characteristics, the four groups were similar in age, sex, aetiology, previous pancreatitis and diabetes. Compared with the severity of the disease in the other groups, the APACHE II scores(9.91 ± 4.65, 9.46 ± 5.05, respectively) and organ failure rate (40.9 and 50%, respectively)were higher in the extrapancreatic infection group and the combined infection group (*P <* 0.05). The frequency of surgical intervention and hospitalization time in patients with NP complicated with extrapancreatic infection was greatly increased (*P <* 0.05). Regarding the primary outcome, patients in the combined infection group had longer hospital stays (68.28 ± 51.80 vs 55.58 ± 36.24, *P <* 0.05) and higher mortality (24.0% vs 9.2%, *P <* 0.05) than patients in the pancreatic infection group. In addition, patients in the extrapancreatic infection group also showed high intensive care utilization (59.1%) and mortality rates (18.2%). Among the 137 AP patients with infection complications, 89 patients exhibited multidrug-resistant (MDR) microorganisms, and the mortality rate of patients with MDR bacterial infection was higher than that of patients with non-MDR bacterial infection (24.7% vs 3.6%, *P =* 0.001).

**Conclusion:**

Clinicians should be aware that extrapancreatic infection (EPI) significantly aggravates the main outcome in pancreatic infection patients. Infection with MDR bacteria is also associated with AP mortality.

## Background

Acute pancreatitis (AP) is a complex inflammatory disease with a highly variable severity [1]. Hospitalizations for AP patients in the United States and Europe have steadily increased over the past decade [2]. AP is a common cause of death of the gastrointestinal tract, and the overall mortality of AP patients is approximately 5% and can reach up to 20-30% in patients with severe acute pancreatitis (SAP) and infected necrosis [3]. AP can be subdivided into interstitial oedematous pancreatitis and necrotizing pancreatitis [4]. The majority of patients have localized or diffuse enlargement of the pancreas due to inflammation and may have some peripancreatic fluid. Symptoms of interstitial oedematous pancreatitis usually resolve within a week [5]. The natural course of pancreatic and peripancreatic necrosis is variable because it can be liquid or solid and sterile or infectious. Pancreatic necrosis and pancreatic fluid collection(s) are sterile in the early stages of the disease, but as many as 40% of patients become infected during the course of the disease [6]. Infection is a major complication that jeopardizes the course of AP [7], and infected necrotizing pancreatitis(INP) is considered to be a determinant of mortality in studies of acute pancreatitis [8]. There are two peaks in the severity of AP; the early peak is followed by a high mortality rate within 2 weeks of the onset of the disease. A cytokine cascade is activated by systemic inflammation, which is clinically manifested as systemic inflammatory response syndrome (SIRS) [9]. When SIRS persists, the risk of organ failure (OF) increases significantly, and the late peak is due to infected necrosis-induced sepsis [10]. Despite improvements in diagnostic and therapeutic techniques, the high mortality rate of SAP has not decreased in the last decade [11]. Clarification of the differential factors in the clinical course and prognosis of AP patients plays an important role in reducing the mortality rate of AP patients. Disruption of the intestinal barrier and displacement of intestinal bacteria in patients with acute pancreatitis have been recognized as the causes of necrotic infection in pancreatitis. Both bacterial and fungal infections can occur intra-abdominally in AP, with primary bacterial infections, particularly of necrotic pancreatic tissue, being the most common [12]. Infections, such as bacteraemia, pneumonia, and urinary tract infection, can also occur in extrapancreatic sites [13]. Moreover, the presence of extrapancreatic complications contributes to morbidity and mortality [14].

EPI is thought to be the translocation of intestinal bacteria into the systemic circulation or an iatrogenic infection acquired through a percutaneous drainage tube, an intravenous catheter, a urinary tube, etc. [15]. Extrapancreatic infections have received increasing attention in recent years, and even after adjusting for risk factors based on severity, they are still associated with increased mortality from the disease. In the course of AP, extrapancreatic infections need to be actively evaluated and treated. Previous studies have reported an increased incidence of infection with NP complicated by bacteraemia or pulmonary infection and an association between acute pancreatitis organ failure and high mortality [16].

This study retrospectively analyzsed differences in the clinical treatments and prognostic parameters between patients with pancreatic and extrapancreatic infections.

## Methods

We retrospectively analyzed the clinical data of 285 patients diagnosed with AP at Tianjin Nankai Hospital between January 2016 and September 2019, mainly composed of patients with moderately severe acute pancreatitis (MSAP) and SAP. The initial diagnoses of these patients were mainly AP, pancreatic pseudocyst, pancreatic abscess, necrotizing pancreatitis (NP), and SAP. We excluded patients with a history of chronic pancreatitis, pancreatic surgery, or biliary, duodenal, or ampulla carcinoma, as well as patients who had no pathogen culture at any site.The study was approved by the Ethics Committee of the Nankai University Nankai Hospital (NKYY_YX_IRB_2018_002_01).

### Definitions

#### AP

The diagnosis of AP was based on the 2012 Atlanta guidelines [4]: 1) typical abdominal irradiated pain; 2)serum amylase and/or lipase at least three times greater than the upper limit of normal; 3)characteristic findings of AP on contrast-enhanced computed tomography (CECT) and, less commonly, magnetic resonance imaging (MRI) or transabdominal ultrasonography. AP was diagnosed if two of the above three characteristics were met. Based on the Atlanta definition, MSAP and SAP depend on the presence of organ and multi-organ failure and local complications.

#### NP

The definition of NP included both pancreatic parenchymal necrosis with and without peripancreatic necrosis and peripancreatic necrosis alone based on contrast-enhanced computed tomography (CECT) [17].

#### Infected necrotizing pancreatitis (INP)

The diagnosis of INP was based on the presence of extraluminal gas on enhanced CT and positive culture from pancreatic necrosis obtained by first puncture or operation [18].

#### EPI

EPI is a common clinical complication of acute pancreatitis and was defined as an infection, such as pneumonia, urinary tract infection, or cholangitis, at any extrapancreatic site during hospitalization [19]. It was diagnosed when AP patients were suspected to have infection symptoms, but there was no clear sign of infection (bubbles) in the imaging of necrotic pancreatic tissue. The necrotic tissue and pus culture of puncture and drainage were negative. Multisite bacterial culture could diagnose or exclude suspected patients with extrapancreatic infection.

#### SIRS

Definition of SIRS proposed by American college of chest physician/Society of Critical Care Medicine Consensus Conference in 1991. More than one of the following clinical manifestations can be diagnosed as SIRS [20]: 1) a body temperature greater than 38 °C or less than 36 °C; 2) a heart rate greater than 90 beats per minute; 3) tachypnea, manifested by a respiratory rate greater than 20 breaths per minute, or hyperventilation, as indicated by a PaCO_2_ of less than 32 mmHg; and 4) WBC count < 4.0 × 10^9^/L or > 12.0 × 10^9^/L or the presence of more than 10% immature neutrophils.

#### OF

OF was defined by the modified Marshall score as greater than 2 points in any respiratory, circulatory, or renal system. If organ failure resolved within 48 h, it was referred to as transient organ failure, and if it exceeded 48 h, it was defined as persistent organ failure (POF) [21]. Acute pancreatitis with POF was defined as severe acute pancreatitis (SAP).

#### MDR bacteria

MDR bacteria, which are bacteria that are usually sensitive to three or more commonly used antibiotics but show simultaneous resistance to these drugs.

### Clinical management of patients

All parameters of the Acute Physiology and Chronic Health Examination (APACHE) II score, SIRS, and the modified Marshall score were recorded within 1 week of admission. Many patients were transferred to our hospital from other hospitals a few days after the onset of the disease, so we recorded the relevant parameters when the patient was admitted to hospital to assess the severity of the disease, including the APACHE II score. All patients were treated at our institution according to a standardized interdisciplinary management protocol. Specific measures included: intensive care, oxygen inhalation, fluid resuscitation to correct fluid losses and maintain adequate intravascular volume, as well as homeostasis of electrolytes and acid base, fasting and gastrointestinal decompression, in addition to an H_2_-blocking agent or proton pump inhibitor, to rest the pancreas. Peripheral and central analgesics were used to relieve pain. A broad spectrum of antibiotics was used for patients suspected of or diagnosed with infection.

For patients suspected of peripancreatic or systemic sepsis (sepsis was defined according to the 1992ACCS/SCCM criteria [22]), we performed CT-guided aspiration. Percutaneous catheter drainage (PCD) was performed at the time of initial aspiration if the fluid appeared turbid, and the fluid and/or samples obtained by the first puncture and drainage were processed for Gram staining and culture. Patients had repeat aspiration performed for persistent sepsis with new or unresolved fluid collections. According to the “step-up approach”, patients with pancreatic infectious necrosis confirmed by culture and unresponsive by PCD were treated surgically. Surgical intervention was delayed for more than 4 weeks and preferably achieved via a minimally invasive technique. In our institution, minimal access retroperitoneal pancreatic necrosectomy (MARPN), small incision pancreatic necrosectomy (SIPN), or single-incision access port retroperitoneoscopic debridement (SIAPRD) were reasonably applied in the following steps according to professional knowledge and appropriate conditions. In our previous studies, there were no significant differences in the primary outcomes for the three different procedures [23]. When AP patients suspected to have infection symptoms, but there was no clear indication of a pancreatic necrosis infection, we performed a multisite culture, targeting blood, pleural fluid, bile, urine, sputum, and catheters. Antibiotics were given when the culture was positive for EPI, such as bacteraemia, pulmonary infection, urinary tract infection and catheter-related infection.

### Subgroups

Culture and sensitivity reports were obtained from various sources, including the percutaneous drainage of peripancreatic fluid, pancreatic necrosis from the surgical intervention, blood, urine, ascites, pleural fluid, and bile, and patients were divided into 4 groups according to the sources of positive culture. The sterile group consisted of patients with negative bacterial culture reports. The pancreatic infection group comprised patients with pancreatic and peripancreatic sources of positive culture (mainly from percutaneous puncture catheter drainage of pancreatic necrosis and peripancreatic fluid, which could also be obtained by surgical intervention). The extrapancreatic infection group included patients with extrapancreatic sources of positive culture (including blood, bile, ascites, pleural effusion, urine, sputum, catheters, etc.), and the combined infection group included patients with positive cultures from both pancreatic and extrapancreatic sites.

### Data collection

We collected the following variables from the medical record during the patient’s hospitalization: baseline characteristics (age, sex, aetiology, BMI, APACHE II score evaluated within 24 h of admission, extent of pancreatic necrosis, etc.), laboratory indexes (white blood cells, lymphocytes, C-reactive protein, albumin, amylase, etc.), and prognostic parameters (length of stay, mortality, surgical intervention, complications, etc.).

### Statistical analysis

Data were expressed as the mean ± standard deviation (SD), percentage or frequency. SAS 9.4 (SAS Institute, Cary, NC, USA) was used for data sorting and statistical analysis, and a variance analysis was used to compare the differences in quantitative data among the four groups. If the differences were significant, the Least-significant Difference (LSD)-t method was used for a pairwise comparison. Chi-squared tests were used to compare differences among the four groups for the qualitative data; if there was a significant difference, further Bonferroni correction methods were applied. Differences with *P <* 0.05 were considered statistically significant.

## Results

The 285 patients were divided into four groups according to the positive culture sources: 148 patients in the sterile group, 65 patients in the pancreatic infection group, 22 patients in the extrapancreatic infection group, and 50 patients in the combined (pancreatic and extrapancreatic) infection group.

### Demographic characteristics

The mean age of the patients was 47.1 ± 15.3 years, and 63.9%(182/285) were male. The mean BMI was 25.69 ± 4.16 kg/m^2^, and there were significant differences in BMI among the different subgroups (26.18 ± 4.10 kg/m^2^ vs 24.77 ± 3.57 kg/m^2^ vs 23.72 ± 4.75 kg/m^2^ vs 26.28 ± 4.39 kg/m^2^, *P =* 0.01). Among the causes of AP, gallstones accounted for 40.3% (115/285), hyperlipidaemia 31.6% (90/285), alcohol 11.6% (33/285), ERCP 1.1% (3/285), and other 15.4% (44/285). Gallstones and hyperlipidaemia were the main aetiologies of AP. There were 101 patients included in the analysis who had a history of diabetes, and 26.0% (74/285) of the patients had a history of AP. Our institution is a tertiary diagnosis and treatment hospital, and 42.8% (122/285) of the patients were transferred to our hospital after treatment in other hospitals. No significant differences in age, sex, aetiology, previous history of pancreatitis and diabetes or transfer were observed among the four groups (Table 1).

**Table 1:** Characteristics of patients in the four groups

	Sterile group (*n =* 148)	Pancreatic infection group (*n =* 65)	Extrapancreatic infection group (*n =* 22)	Combined infection group (*n =* 50)	Total (*n =* 285)	P*
Age [year, mean ± SD]	45.79 ± 14.95	47.94 ± 14.62	51.00 ± 16.72	48.16 ± 16.50	47.10 ± 15.29	0.401
Gender [*n*(%)]						
Male	92(62.2)	42(64.6)	15(68.2)	33(66.0)	182(63.9)	0.924
Female	56(37.8)	23(35.4)	7(31.8)	17(34.0)	103(36.1)	
BMI [kg/m^2^, mean ± SD]	26.18 ± 4.10^bc^	24.77 ± 3.57^a^	23.72 ± 4.75^ad^	26.28 ± 4.39^c^	25.69 ± 4.16	0.010
Etiology [*n*(%)]						
Gallstones	51(34.5)	31(47.7)	13(59.1)	20(40.0)	115(40.3)	0.551
Hyperlipidemia	54(36.5)	15(23.1)	5(22.7)	16(32.0)	90(31.6)	
Alcohol abuse	19(12.8)	6(9.2)	1(4.6)	7(14.0)	33(11.6)	
ERCP	1(0.7)	1(1.5)	0(0.0)	1(2.0)	3(1.1)	
Other	23(15.5)	12(18.5)	3(13.6)	6(12.0)	44(15.4)	
Previous pancreatitis [*n*(%)]	44(29.7)	15(23.1)	3(13.6)	12(24.0)	74(26.0)	0.360
Diabetes [*n*(%)]	55(37.2)	22(33.8)	5(22.7)	19(38.0)	101(35.4)	0.580
Transfer[*n*(%)]	65(43.9)	27(41.5)	9(40.9)	21(42.0)	122(42.8)	0.983
Disease severity						
APACHE II scores [mean ± SD]	7.27 ± 4.96^cd^	6.57 ± 4.20^cd^	9.91 ± 4.65^ab^	9.46 ± 5.05^ab^	7.70 ± 4.90	0.001
SIRS [*n*(%)]	87(58.8)	30(46.2)	13(59.1)	35(70.0)	165(57.9)	0.081
Organ failure [*n*(%)]	23(15.5)^cd^	16(24.6)^d^	9(40.9)^a^	25(50)^ab^	73(9.8)	< 0.001

*BMI* Body mass index, *ERCP* Endoscopic retrograde cholangiopancreatography, *APACHE* Acute Physiology and Chronic Health Evaluation, *SIRS* Systemic inflammatory response syndrome

* *P* value is the statistical value of ANOVA

^a^ Significant difference compared with the sterile group; ^b^ significant difference compared with the pancreatic infection group; ^c^ significant difference compared with the extrapancreatic infection group; ^d^ significant difference compared with the combined infection group, *P* <0.05 represents a statistically significant difference

### Severity of acute pancreatitis at admission

The APACHE II score was evaluated within 24 h after admission, and scores greater than 8 were defined as SAP. The scores of the extrapancreatic infection group and the combined infection group (9.91 ± 4.65 and 9.46 ± 5.05, respectively) were higher than those of the sterile group and the pancreatic infection group (7.27 ± 4.96 and 6.57 ± 4.20, respectively; *P <* 0.05). We also observed whether SIRS or OF occurred in patients at the early stage of admission and recorded these parameters, which were important references for the early intensification of AP. Among the four groups, there were significant differences in organ failure among the four groups (15.5% vs 24.6% vs 40.9% vs 50.0%; *P <* 0.001),the incidence of OF in the combined infection group(50%) was higher than that in the sterile group and pancreatic infection group (15.5% and 24.6%, respectively; *P <* 0.05).

### Laboratory investigation

We recorded the laboratory data of 285 patients, including blood cell count, liver and kidney function, electrolytes, coagulation function and other indicators. Differences were found among the four groups in regard to the WBC(13.65 ± 5.81 × 10^9^/L vs 11.57 ± 4.9 × 10^9^/L vs 11.41 ± 5.99 × 10^9^/L vs 14.41 ± 5.65 × 10^9^/L; *P* = 0.013), HCT(40.44 ± 8.34% vs 36.81 ± 7.53% vs 36.65 ± 11.06% vs 36.23 ± 9.15%; *P* = 0.002), PCT(3.34 ± 5.2 ng/mL vs 6.12 ± 8.15 ng/mL vs 6.90 ± 9.12 ng/mL vs 12.70 ± 23.95 ng/mL; *P* < 0.001), APTT(28.17 ± 6.69 s vs 29.22 ± 9.15 s vs 33.10 ± 7.66 s vs 31.79 ± 17.69 s; *P* = 0.048), and serum creatinine parameters (104.6 ± 108.7umol/L vs 80.57 ± 49.90umol/L vs 135.0 ± 157.8umol/L vs 148.7 ± 173.0umol/L; *P* = 0.014). No significant differences were found in other laboratory indicators (Table 2).

**Table 2:** Laboratory indicators by group [mean ± SD]

	Sterile group (*n =* 148)	Pancreatic infection group (*n =* 65)	Extrapancreatic infection group (*n =* 22)	Combined infection group (*n =* 50)	Total (*n =* 285)	P*
WBC (×10^9^/L)	13.65 ± 5.81^b^	11.57 ± 4.90^ad^	11.41 ± 5.99^d^	14.41 ± 5.50^bc^	13.14 ± 5.65	0.013
HCT (%)	40.44 ± 8.34^bd^	36.81 ± 7.53^a^	36.65 ± 11.06	36.23 ± 9.15^a^	38.57 ± 8.73	0.002
CRP (mg/L)	122.2 ± 100.6	87.67 ± 81.51	97.74 ± 69.34	117.4 ± 83.05	111.6 ± 92.14	0.070
PCT (ng/mL)	3.34 ± 5.24^d^	6.12 ± 8.15	6.90 ± 9.12	12.70 ± 23.95^a^	5.83 ± 12.07	< 0.001
ALB (g/L)	35.79 ± 7.24	34.72 ± 6.50	34.26 ± 7.10	34.55 ± 7.71	35.21 ± 7.14	0.554
TBIL (μmol/L)	32.10 ± 58.94	36.50 ± 72.86	44.90 ± 45.50	40.32 ± 39.82	35.54 ± 58.64	0.703
ALT (U/L)	69.95 ± 132.4	60.12 ± 99.49	142.2 ± 245.3	106.1 ± 164.2	79.63 ± 144.8	0.054
AST (U/L)	54.97 ± 99.91	50.82 ± 67.85	84.95 ± 195.8	103.8 ± 200.2	64.90 ± 127.9	0.076
AMS (U/L)	425.3 ± 725.8	375.9 ± 568.0	651.0 ± 1033	598.3 ± 898.9	461.8 ± 756.3	0.242
APTT (s)	28.17 ± 6.69^cd^	29.22 ± 9.15	33.10 ± 7.66^a^	31.79 ± 17.69^a^	29.42 ± 10.16	0.048
FIB (g/L)	4.83 ± 1.85	4.52 ± 1.90	4.38 ± 2.18	4.16 ± 1.75	4.61 ± 1.88	0.146
BUN(mmol/L)	7.43 ± 5.84	6.79 ± 4.18	8.54 ± 11.20	9.59 ± 7.04	7.75 ± 6.35	0.094
Serum creatinine (umol/L)	104.6 ± 108.7^d^	80.57 ± 49.90^d^	135.0 ± 157.8	148.7 ± 173.0^ab^	109.2 ± 119.2	0.014
Serum calcium (mmol/L)	1.81 ± 0.38	4.02 ± 16.38	1.81 ± 0.47	2.57 ± 4.92	2.45 ± 8.09	0.319

*WBC* White blood cell, *HCT* Hematocrit, *CRP* C-reactive protein, *PCT* Procalcitonin, *ALB* Albumin, *TBIL* Total bilirubin, *ALT* Alanine aminotransferase, *AST* Aspartate aminotransferase, *AMS* Serum amylase, *APTT* Activated partial thromboplastin time, *FIB* Fibrinogen, *BUN* Blood urea nitrogen

* *P* value is the statistical value of ANOVA

^a^ Significant difference compared with the sterile group; ^b^ significant difference compared with the pancreatic infection group; ^c^ significant difference compared with the extrapancreatic infection group; ^d^ significant difference compared with the combined infection group, *P* <0.05 represents a statistically significant difference

### Characteristics of infection

**Fig. 1:**
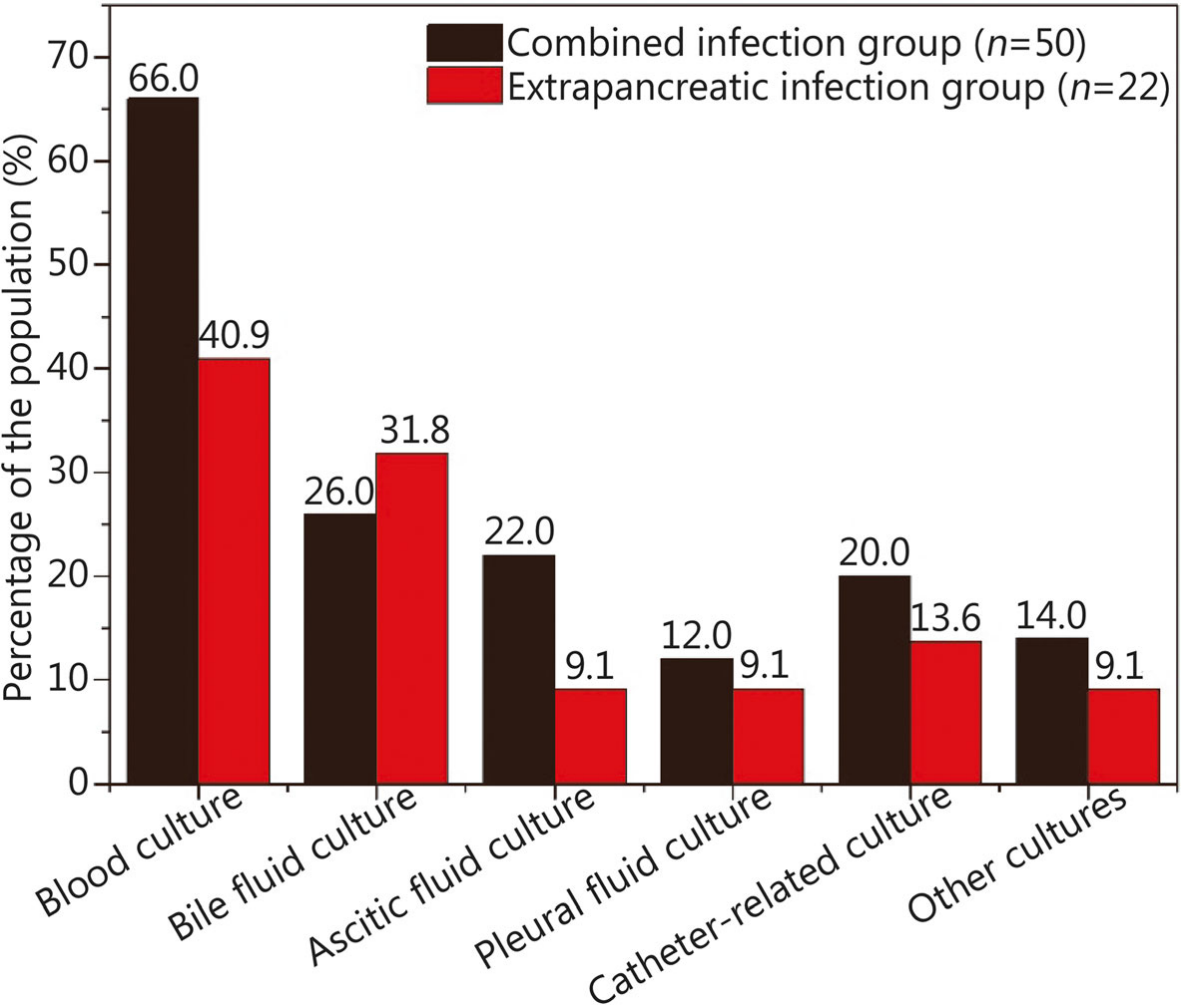
Culture sources of extrapancreatic infection and combined infection groups

All positive culture reports were included in the analysis, and we analyzed the microbiological profile and timeline of patients in the three infected groups. According to the culture results, the majority of patients had polymicrobial infections. Table 3 shows the pathogens isolated in 137 patients (including the pancreatic infection group, the extrapancreatic infection group, and the combined infection group).We use mortality as an indicator of disease severity. *Klebsiella spp*, *Escherichia coli*, *Acinetobacter spp* were the main Gram-negative organism identified, and Gram-positive bacteria are mainly *Enterococcus spp*, *Staphylococcus spp*. In the above specific organisms, we performed statistical analysis with no significant difference in mortality. Twelve patients found fungal infection, mainly candida infection. In our study, there was still no difference in mortality between bacterial infection and fungal infection. In 137 patients with AP complicated with infection, 59.1% (81/137) of patients had MDR bacterial infection, and the mortality rate of MDR bacterial infection was higher than that of non-MDR bacterial infections (24.7% vs 3.6%, *P =* 0.001). Extrapancreatic infections consisted mainly of bacteraemia and biliary tract infection, the site of extrapancreatic infection is also shown in Fig. 1. The mortality rate of patients with bacteraemia was higher than that of patients with infections in other sites (31.0% vs 10%, *P =* 0.046).

**Table 3:** Culture results in different infection groups [*n*(%)]

	Pancreatic infection (65 positive)	Extrapancreatic infection (22 positive)	Combined infection (50 positive)
**Gram-negative**			
*Klebsiella spp.*	12(18.5)	5(22.7)	24(48.0)
*Escherichia coli*	14(21.5)	2(9.1)	22(44.0)
*Acinetobacter spp.*	11(16.9)	3(13.6)	15(30.0)
*Enterobacter spp.*	8(12.3)	2(9.0)	10(20.0)
*Pseudomonas spp.*	6(9.2)	0(0.0)	6(12.0)
Others	11(16.9)	4(18.2)	19(38.0)
**Gram-positive**			
*Enterococcus spp.*	26(40.0)	10(45.5)	39(78.0)
*Staphylococcus spp.*	30(46.2)	5(22.7)	36(72.0)
*Corynebacterium spp.*	5(7.7)	0(0.0)	5(10.0)
*Streptococcus spp.*	3(4.6)	1(4.5)	5(10.0)
Others	1(1.5)	0(0.0)	2(4.0)
**Fungi**			
*Candida spp.*	3(4.6)	2(9.1)	6(12.0)
MDR bacterial infection	35(53.8)	8(36.4)	38(76.0)

*MDR* Multidrug resistant

We performed a preliminary analysis of the time of infection in the pancreatic infection group, the extrapancreatic infection group, and the combined infection group. The time from onset to the first discovery of positive culture results in the three groups was 28.57 ± 20.18, 22.06 ± 11.52 and 23.36 ± 14.72 days, respectively. According to the clinical records, 19 of the 50 patients in combined infection group first developed extrapancreatic infection, 21 patients had pancreatic infection, and 10 patients had pancreatic and extrapancreatic infection in the same period. Forty-two percent of patients with extrapancreatic infection had microorganisms isolated similar to peripancreatic-isolated microorganisms. Due to the complexity of clinical treatment, there may be some deviation in the type and quantity of pathogenic bacteria.

### Clinical treatment

In terms of nutritional support, there were differences between the four groups, but subgroup analysis showed no difference between the three groups of infected patients. Mechanical ventilation was performed on 29.5% (84/285) of patients. The utilization rate of mechanical ventilation in the combined infection group (50.0%) and the extrapancreatic infection group (54.5%) was significantly higher than that in the other groups (19.6 and 27.7%) and the difference was significant between the subgroups (*P <* 0.05). The duration of antibiotics among the four groups was significantly different (9.53 ± 6.04 vs 22.34 ± 13.56 vs 17.32 ± 20.12 vs 40.32 ± 32.06; *P <* 0.001), and the duration of antibiotics in the pancreatic infection group and the combined infection group were longer than that in the other two groups. The extent of pancreatic necrosis in the pancreatic infection group and the combined infection group were greater than that in the other groups, the incidence of infection complications was lower in patients with a lesser degree of necrosis (< 30.0%) than in patients with extensive necrosis (> 30%) (23.1% vs 76.9%, 20% vs 80%). The frequency of PCD treatment and surgical debridement was also significantly increased (*P <* 0.001, Table 4).

**Table 4:** Clinical management comparison by group

	Sterile group (*n =* 148)	Pancreatic infection group (*n =* 65)	Extrapancreatic infection group (*n =* 22)	Combined infection group (*n =* 50)	Total (*n =* 285)	P*
Nutritional support [*n*(%)]						
Parenteral nutrition only	89(60.1)^bd^	17(26.2)^a^	8(36.4)	6 (12)^a^	120(42.1)	< 0.001
Enteral and parenteral nutrition	53(35.8)^bd^	43(66.2)^a^	14(63.6)	41(82.0)^a^	151(53.0)	< 0.001
Antibiotics duration [d, mean ± SD]	9.53 ± 6.04^bd^	22.34 ± 13.56^ad^	17.32 ± 20.12^d^	40.32 ± 32.06^abc^	18.46 ± 19.92	< 0.001
Mechanical ventilation[*n*(%)]	29(19.6)^cd^	18(27.7)^cd^	12(54.5)^ab^	25(50.0)^ab^	84(29.5)	< 0.001
Extent of necrosis[*n*(%)]						
< 30%	99(66.9)^bd^	15(23.1)^ac^	14(63.6)^bd^	10(20)^ac^	138(48.4)	< 0.001
30-50%	31(20.9)	23(35.4)	5(22.7)	16(32)	75(26.3)	
> 50%	18(12.2)	27(41.5)	3(13.7)	24(48)	72(25.3)	
Surgical intervention(times/case)						
PCD	0.97 ± 0.84^bd^	1.95 ± 1.37^ac^	0.86 ± 1.04^bd^	2.38 ± 1.74^ac^	1.44 ± 1.32	< 0.001
Surgical debridement^*^	0.07 ± 0.57^bd^	0.83 ± 1.34^ac^	0.18 ± 0.85^bd^	1.14 ± 1.97^ac^	0.44 ± 1.22	< 0.001

*PCD* Percutaneous catheter drainage. ^*^Surgical debridement: Including minimal access retroperitoneal pancreatic necrosectomy (MARPN), small incision pancreatic necrosectom (SIPN), or single-incision access port retroperitoneoscopic debridement (SIAPRD)

* *P* value is the statistical value of ANOVA

^a^ Significant difference compared with the sterile group; ^b^ significant difference compared with the pancreatic infection group; ^c^ significant difference compared with the extrapancreatic infection group; ^d^ significant difference compared with the combined infection group, *P* <0.05 represents a statistically significant difference

### Primary and secondary outcomes

We used hospital stay and mortality as the major outcome measures, and Table 5 shows the subgroup analysis of intensive care utilization, hospitalization, surgical complications, and mortality. The average length of stay in four groups were 21.95 ± 24.59 d, 55.58 ± 36.24 d, 31.27 ± 31.06 d, and 68.28 ± 51.80 d, respectively. A pairwise comparison of the mean length of hospital stay across patient subgroups was also performed, and the hospital stay in the combined infection group was significantly different from that in the other groups (*P <* 0.05). The mortality rates noted in four groups were 6.1, 9.2, 18.2, and 24.0%, respectively. Mortality was highest in the combined infection group, followed by that in the extrapancreatic infection group. The comparisons showed that the mortality of the combined infection group was different from that of the sterile group and the pancreatic infection group(*P <* 0.05). Intensive care was received by 38.6% of the patients, the intensive care utilization rate in the extrapancreatic infection group (59.1%) and the combined infection group (60.0%) was higher than that in the other groups, and the difference was significant (*P <* 0.05). Of the 285 patients, there were 36 patients with surgical complications, 25 patients with abdominal cavity or gastrointestinal bleeding, 7 patients with gastrointestinal fistula, and 4 patients with pancreatic encephalopathy. Complications occurred in 18 patients in the combined infection group, 4 patients in the sterile group, 10 patients in the pancreatic infection group, and 4 patients in the extrapancreatic infection group. The incidence of complications in combined infection group was 36.0%(18/50), which was higher than that in other groups (2.7, 15.4, and 18.1%, respectively). The complications were analyzed in the different subgroups, and there were significant differences in the combined infection group vs the sterile group (*P <* 0.05) and the combined infection group vs pancreatic infection group (*P <* 0.05).

**Table 5:** Comparison of clinical outcomes among the four group

	Sterile group (*n =* 148)	Pancreatic infection group (*n =* 65)	Extrapancreatic infection group (*n =* 22)	Combined infection group (*n =* 50)	Total (*n =* 285)	P*
Intensive care utilization [*n*(%)]	40(27.0)^bcd^	27(41.5)^ad^	13(59.1)^a^	30(60.0)^ab^	110(38.6)	< 0.001
Hospital stay [d mean ± SD]	21.95 ± 24.59^bd^	55.58 ± 36.24^acd^	31.27 ± 31.06^bd^	68.28 ± 51.80^abc^	38.45 ± 38.91	< 0.001
Surgical complications [*n*(%)]						
None	144(97.3)^bcd^	55(84.6)^ad^	18(81.8)^a^	32(64.0)ab	249(87.4)	< 0.001
Intraabdominal hemorrhage	4(2.7)	5(7.7)	3(13.6)	13(26.0)	25(8.8)	
Gastrointestinal fistula	0(0.0)	4(6.2)	0(0.0)	3(6.0)	7(2.5)	
Pancreatic encephalopathy	0(0.0)	1(1.5)	1(4.5)	2(4.0)	4(1.4)	
Mortality [*n*(%)]	9(6.1)^cd^	6(9.2)^d^	4(18.2)^a^	12(24.0)^ab^	31(10.9)	0.003

* *P* value is the statistical value of ANOVA

^a^ Significant difference compared with the sterile group; ^b^ significant difference compared with the pancreatic infection group; ^c^ significant difference compared with the extrapancreatic infection group; ^d^ significant difference compared with the combined infection group, *P* <0.05 represents a statistically significant difference

## Discussion

In patients with AP, more than 80% of deaths are attributed to septic complications as a consequence of bacterial infection in pancreatic necrosis [24]. We observed an increased mortality rate in patients with infection at both pancreatic and extrapancreatic sites or at extrapancreatic sites alone (24.0 and 18.2%, respectively). The occurrence of EPI is also thought to be associated with the morbidity and mortality of pancreatitis [25]. EPI, such as bloodstream infections, pneumonia, and urinary tract infections, occur early in up to 24.0% of patients with AP and can double the mortality rate [26]. In our analysis, the incidence of EPI was 25.3%(72/285), which was close to that in the above study.

In a prospective study of AP in a large sample size, patients in the sterile necrosis group had a lower CT severity index, more systemic complications, and a smaller extent of pancreatic necrosis than patients with infected necrosis [27]. The results of our study are similar, and the incidence of infection complications was lower in patients with a lesser degree of necrosis (< 30%) than in patients with extensive necrosis (> 30%). During the natural course of AP, persistent SIRS increases the risk of organ failure [28]. In our study, the incidence of SIRS in the extrapancreatic infection group and combined infection group was positively correlated with the incidence of OF (40.9 and 50.0%, respectively), and both groups showed increased mortality (18.2 and 24.0%, respectively). Moreover, the APACHE II scores were significantly increased in both groups. Due to the occurrence of OF, the mortality rate of the sterile group was 6.1%(9/148).

The most common pathogenesis of pancreatic infection is intestinal bacterial translocation. It has been reported that monomicrobial infection is more common than polymicrobial infection in pancreatic infectious necrosis [29]. However, in this study, polymicrobial infection was common in both the pancreatic infection group and the combined infection group. Our hospital accepts patients transferred from other hospitals, and we think that polymicrobial infection may be related to the possibility of secondary iatrogenic infection.

Sainio et al. [30] believe that EPI is closely related to pancreatic necrosis. There is also evidence [31] from the field of bacteriology that extrapancreatic infection accelerates infectious pancreatic necrosis. Additional studies [32] have shown that EPI occurs before pancreatic infection. Elizabeth et al. [33] found that pulmonary infection in INP patients usually occurred earlier than INP, and the patients had the same pathogen in the bronchial site and pancreatic necrosis. Multivariate analysis showed that lung infection was associated with mortality and INP. This event could be related to respiratory failure in SAP and admission to the intensive care unit, usually under mechanical ventilation. In this study, the incidence of OF (27.3%) and the utilization rate of mechanical ventilation (54.5%) in the extrapancreatic infection group and combined infection group were increased. At the same time, mortality in both groups increased significantly.

Patients with AP require early aggressive fluid resuscitation to maintain haemodynamic stability, and patients also need adequate analgesia to eliminate or markedly reduce pain. In a prolonged period of “nothing by mouth”, nutritional support is an important part of treatment until any nausea and vomiting have been overcome. The concept of pancreatic rest is well known in the management of AP. Total parental nutrition (TPN) is considered to be nutritional support without stimulating the pancreas and potentially aggravating the disease. In recent years, several studies have shown that intestinal barrier atrophy is related to bacterial overgrowth and can easily lead to bacterial translocation and SIRS, increasing the risk of infected pancreatic necrosis. The use of parenteral nutrition (PN) is harmful to patients with SAP with intestinal barrier destruction [34, 35]. Additional evidence [36–39] suggests that the cost of enteral nutrition (EN) is lower than that of TPN. The cost advantage and the improvement of some important outcomes have led to a general change in EN in patients with AP. In our data, the use of PN or EN alone and in combination was determined on the basis of the disease itself. Oral food intake in patients with mild pancreatitis usually recovers within 1 week of hospitalization, nutritional support is not required, and the degree of disease in the sterile group was mild; the proportion of patients treated with TPN alone accounted for 60.1%(89/148). In the early stage of SAP, with severe abdominal pain, nausea and vomiting and a systemic inflammatory response, we prefer to manage patients with TPN. When the symptoms of the patient are eliminated and nutrition is not available for a long time, we performed intestinal feeding as early as possible to reduce the risk of pancreatic necrosis. The limitation of EN is that some patients cannot tolerate the mechanical discomfort of a nasojejunal or nasogastric tube. In the case of the above, we considered TPN treatment. Thus, nutritional support must be tailored to the individual patient and modified depending on the patient’s response and tolerance. Based on the above theory, in the infected group, we are more likely to choose treatment with EN combined with PN.

Pancreatic infectious necrosis has been regarded as an indication for surgical intervention because of its fatal outcomes. Large sterile areas may become infected during the course of the disease; if untreated, infected pancreatic necrosis could result in high mortality. Sterile necrosis is best given medical management in the first 2-3 weeks; if abdominal pain persists and prevents oral intake, we consider surgical debridement. The treatment of NP has experienced a shift from open surgery to minimally invasive techniques. Recent multicentre studies, randomized trials, evidence-based guidelines, and consensus statements have endorsed the safety and efficacy of endoscopic and other minimally invasive techniques for the treatment of NP [40, 41]. In our institution, timely surgical intervention in patients with pancreatic infection reduced mortality, whereas in NP patients with extrapancreatic infection, the disease was severe, surgical complications were numerous, and mortality was high. The incidence of OF in the extrapancreatic infection group and the combined infection group was 40.9%(9/22) and 50.0%(25/50), respectively, which was significantly higher than that in the pancreatic infection group (24.6%), and the same trend also appeared for mortality. Patients with extrapancreatic and combined infections are the most severe in the early stages, and the emergence of MDR bacteria may be one of the causes of high mortality. Prompt surgical intervention may have accounted for the low mortality in the pancreatic infection alone group. The frequency of surgical intervention in the combined infection group was higher than that in the pancreatic infection group; the incidence of surgical complications, such as abdominal bleeding, was much higher than that in the pancreatic infection group; and the hospitalization time in the combined infection group was the longest among the four groups. EPI significantly aggravates the main outcome in patients with acute necrotizing pancreatitis.

In addition, this retrospective study has some limitations. Retrospective analysis relies on the review of clinical data, which can be biased in a study. This study is based on a single centre, and outcomes in this patient series, especially the mortality rate, may not be applicable to every institution. As a diagnosis and treatment centre for hepatobiliary and pancreatic diseases, our hospital also receives patients treated through other hospitals, which has an impact on the source of the infection, the time of the infection, and the clinical prognosis of the patient. Second, due to the complexity of clinical treatment, there may be some deviation in the type and quantity of pathogenic bacteria. Third, the number of patients with EPI in this study is small, and we will further expand the sample size, with more powerful and larger prospective studies to verify the current results.

## Conclusions

INP combined with extrapancreatic infection significantly increases the incidence of complications and the mortality of pancreatitis. Early detection and control of EPI and MDR bacteria should also be an important part of the clinical management of acute pancreatitis. When AP patients have clinical symptoms of suspected infection, but there is no clear sign of pancreatic infection, priority can be given to the culture and treatment of EPI.

## Data Availability

The datasets generated and/ or analyzed during the current study are available from the corresponding author upon reasonable request.

## Abbreviations

AP: Acute pancreatitis
APACHE II: Acute physiology and chronic health evaluation
APTT: Activated partial thromboplastin time
BMI: Body mass index
CECT: Contrast-enhanced computed tomography
EN: Enteral nutrition
ERCP: Endoscopic retrograde cholangio-pancreatography
EPI: Extrapancreatic infection
HCT: Hematocrit
INP: Infected necrotizing pancreatitis
LSD: Least-significant Difference
MARPN: Minimal access retroperitoneal pancreatic necrosectomy
MDR: Multidrug-resistant
MRI: Magnetic resonance imaging
MSAP: Moderately severe acute pancreatitis
NP: Necrotizing pancreatitis
OF: Organ failure
PCD: Percutaneous catheter drainage
POF: Persistent organ failure
PCT: Procalcitonin
PN: Parenteral nutrition
SAP: Severe acute pancreatitis
SD: Standard deviation
SIAPRD: Single-incision access port retroperitoneoscopic debridement
SIPN: Small incision pancreatic necrosectom
SIRS: Systemic inflammatory response syndrome
WBC: White blood cell

## References

[1] Staubli SM, Oertli D, Nebiker CA (2015). Laboratory markers predicting severity of acute pancreatitis. Crit Rev Clin Lab Sci.

[2] Petrov MS, Mcilroy K, Grayson L, Phillips AR, Windsor JA (2013). Early nasogastric tube feeding versus nil per os in mild to moderate acute pancreatitis: a randomized controlled trial. Clin Nutr.

[3] Pavlidis P, Crichton S, Lemmich Smith J, Morrison D, Atkinson S, Wyncoll D, et al. Improved outcome of severe acute pancreatitis in the intensive care unit. Crit Care Res Pract. 2013;2013:897107. 10.1155/2013/897107 PMC3594930 23662207

[4] Banks PA, Bollen TL, Dervenis C, Gooszen HG, Johnson CD, Sarr MG (2013). Classification of acute pancreatitis--2012: revision of the Atlanta classification and definitions by international consensus. Gut.

[5] Wu BU, Banks PA (2013). Clinical management of patients with acute pancreatitis. Gastroenterology.

[6] Uhl W, Warshaw A, Imrie C, Bassi C, Mckay CJ, Lankisch PG (2002). IAP guidelines for the surgical management of acute pancreatitis. Pancreatology.

[7] Moka P, Goswami P, Kapil A, Xess I, Sreenivas V, Saraya A (2018). Impact of antibiotic-resistant bacterial and fungal infections in outcome of acute pancreatitis. Pancreas.

[8] Petrov MS, Shanbhag S, Chakraborty M, Phillips AR, Windsor JA (2010). Organ failure and infection of pancreatic necrosis as determinants of mortality in patients with acute pancreatitis. Gastroenterology.

[9] Johnson C, Abu-Hilal M (2004). Persistent organ failure during the first week as a marker of fatal outcome in acute pancreatitis. Gut.

[10] Phillip V, Steiner JM, Algül H (2014). Early phase of acute pancreatitis: assessment and management. World J Gastrointest Pathophysiol.

[11] Yang ZW, Meng XX, Xu P (2015). Central role of neutrophil in the pathogenesis of severe acute pancreatitis. J Cell Mol Med.

[12] Trikudanathan G, Navaneethan U, Vege SS (2011). Intra-abdominal fungal infections complicating acute pancreatitis: a review. Am J Gastroenterol.

[13] Bakker OJ, Van Santvoort H, Besselink MG, Boermeester MA, Van Eijck C, Dejong K (2013). Extrapancreatic necrosis without pancreatic parenchymal necrosis: a separate entity in necrotising pancreatitis?. Gut.

[14] Brown LA, Hore TA, Phillips AR, Windsor JA, Petrov MS (2014). A systematic review of the extra-pancreatic infectious complications in acute pancreatitis. Pancreatology.

[15] Wu BU, Johannes RS, Kurtz S, Banks PA (2008). The impact of hospital-acquired infection on outcome in acute pancreatitis. Gastroenterology.

[16] Besselink MG, Van Santvoort HC, Boermeester MA, Nieuwenhuijs VB, Van Goor H, Dejong CH (2009). Timing and impact of infections in acute pancreatitis. Br J Surg.

[17] Wang M, Wei A, Guo Q, Zhang Z, Lu H, Li A (2016). Clinical outcomes of combined necrotizing pancreatitis versus extrapancreatic necrosis alone. Pancreatology.

[18] Lu JD, Cao F, Ding YX, Wu YD, Guo YL, Li F (2019). Timing, distribution, and microbiology of infectious complications after necrotizing pancreatitis. World J Gastroenterol.

[19] Jain S, Mahapatra SJ, Gupta S, Shalimar, Garg PK (2018). Infected pancreatic necrosis due to multidrug-resistant organisms and persistent organ failure predict mortality in acute pancreatitis. Clin Transl Gastroenterol.

[20] Bone RC, Balk RA, Cerra FB, Dellinger RP, Fein AM, Knaus WA (1992). Definitions for sepsis and organ failure and guidelines for the use of innovative therapies in sepsis. Chest.

[21] Lytras D, Manes K, Triantopoulou C, Paraskeva C, Delis S, Avgerinos C (2008). Persistent early organ failure: defining the high-risk group of patients with severe acute pancreatitis?. Pancreas.

[22] Knaus WA, Sun X, Nystrom O, Wagner DP (1992). Evaluation of definitions for sepsis. Chest.

[23] Hu Y, Jiang X, Li C, Cui Y (2019). Outcomes from different minimally invasive approaches for infected necrotizing pancreatitis. Medicine (Baltimore).

[24] Maher MM, Lucey BC, Gervais DA, Mueller PR (2004). Acute pancreatitis: the role of imaging and interventional radiology. Cardiovasc Intervent Radiol.

[25] Xue P, Deng LH, Zhang ZD, Yang XN, Wan MH, Song B (2009). Infectious complications in patients with severe acute pancreatitis. Dig Dis Sci.

[26] Lankisch PG, Apte M, Banks PA (2015). Acute pancreatitis. Lancet.

[27] Van Santvoort HC, Bakker OJ, Bollen TL, Besselink MG, Ahmed Ali U, Schrijver AM (2011). A conservative and minimally invasive approach to necrotizing pancreatitis improves outcome. Gastroenterology.

[28] Guo Q, Li A, Xia Q, Liu X, Tian B, Mai G (2014). The role of organ failure and infection in necrotizing pancreatitis: a prospective study. Ann Surg.

[29] Garg PK, Khanna S, Bohidar NP, Kapil A, Tandon RK (2001). Incidence, spectrum and antibiotic sensitivity pattern of bacterial infections among patients with acute pancreatitis. J Gastroenterol Hepatol.

[30] Sainio V, Kemppainen E, Puolakkainen P, Haapiainen R, Schröder T, Kivilaakso E (1995). Early antibiotic treatment in acute necrotising pancreatitis. Lancet.

[31] Israil A, Palade R, Chifiriuc M, Vasile D, Grigoriu M, Voiculescu D (2011). Spectrum, antibiotic susceptibility and virulence factors of bacterial infections complicating severe acute pancreatitis. Chirurgia (Bucur).

[32] Bourgaux JF, Defez C, Muller L, Vivancos J, Prudhomme M, Navarro F (2007). Infectious complications, prognostic factors and assessment of anti-infectious management of 212 consecutive patients with acute pancreatitis. Gastroenterol Clin Biol.

[33] Pando E, Alberti P, Hidalgo J, Vidal L, Dopazo C, Caralt M, et al. The role of extra-pancreatic infections in the prediction of severity and local complications in acute pancreatitis. Pancreatology. 2018. 10.1016/j.pan.2018.05.481. 10.1016/j.pan.2018.05.481 29802078

[34] Li JY, Yu T, Chen GC, Yuan YH, Zhong W, Zhao LN (2013). Enteral nutrition within 48 hours of admission improves clinical outcomes of acute pancreatitis by reducing complications: a meta-analysis. PLoS One.

[35] Petrov MS, Whelan K (2010). Comparison of complications attributable to enteral and parenteral nutrition in predicted severe acute pancreatitis: a systematic review and meta-analysis. Br J Nutr.

[36] Marik PE, Zaloga GP (2004). Meta-analysis of parenteral nutrition versus enteral nutrition in patients with acute pancreatitis. BMJ..

[37] Quan H, Wang X, Guo C (2011). A meta-analysis of enteral nutrition and total parenteral nutrition in patients with acute pancreatitis. Gastroenterol Res Pract.

[38] Al-Omran M, Albalawi ZH, Tashkandi MF, Al-Ansary LA. Enteral versus parenteral nutrition for acute pancreatitis. Cochrane Database Syst Rev. 2010. 10.1002/14651858. 10.1002/14651858.CD002837.pub2 PMC7120370 20091534

[39] Eckerwall GE, Axelsson JB, Andersson RG (2006). Early nasogastric feeding in predicted severe acute pancreatitis: a clinical, randomized study. Ann Surg.

[40] Freeman ML, Werner J, Van Santvoort HC, Baron TH, Besselink MG, Windsor JA (2012). Interventions for necrotizing pancreatitis: summary of a multidisciplinary consensus conference. Pancreas.

[41] Trikudanathan G, Attam R, Arain MA, Mallery S, Freeman ML (2014). Endoscopic interventions for necrotizing pancreatitis. Am J Gastroenterol.

